# Quantitative trait loci for large sink capacity enhance rice grain yield under free-air CO_2_ enrichment conditions

**DOI:** 10.1038/s41598-017-01690-8

**Published:** 2017-05-12

**Authors:** Hiroshi Nakano, Satoshi Yoshinaga, Toshiyuki Takai, Yumiko Arai-Sanoh, Katsuhiko Kondo, Toshio Yamamoto, Hidemitsu Sakai, Takeshi Tokida, Yasuhiro Usui, Hirofumi Nakamura, Toshihiro Hasegawa, Motohiko Kondo

**Affiliations:** 1 NARO Institute of Crop Science, 2-1-2 Kannondai, Tsukuba, Ibaraki 305-8602 Japan; 2 NARO Kyushu Okinawa Agricultural Research Center, 496 Izumi, Chikugo, Fukuoka 833-0041 Japan; 3 NARO Central Region Agricultural Research Center, 1-2-1 Inada, Jyoetsu, Niigata 943-0193 Japan; 4 Japan International Research Center for Agricultural Sciences, 1-1 Ohwashi, Tsukuba, Ibaraki 305-8686 Japan; 5 NARO Institute for Agro-Environmental Sciences, 3-1-3 Kannondai, Tsukuba, Ibaraki 305-8604 Japan; 6 NARO Hokkaido Agricultural Research Center, 9-4 Shinseiminami, Memuro-cho, Kasai-gun, Hokkaido 082-0081 Japan; 7 Taiyo Keiki Co. Ltd., 1-12-3 Nakajujo, Kita-ku, Tokyo 114-0032 Japan; 8 NARO Tohoku Agricultural Research Center, 4 Shimokuriyagawaazaakahira, Morioka, Iwate 020-0198 Japan; 9 Graduate School of Bioagricultural Sciences, Nagoya University, Furo-cho, Chikusa-ku, Nagoya, Aichi 464-8601 Japan

## Abstract

The global atmospheric CO_2_ concentration has been increasing annually. To determine the trait that effectively increases rice (*Oryza sativa* L.) grain yield under increased atmospheric CO_2_ concentrations, as predicted in the near future, we grew a chromosome segment substitution line (CSSL) and a near-isogenic line (NIL) producing high spikelet numbers per panicle (CSSL-*GN1* and NIL-*APO1*, respectively) under free-air CO_2_ enrichment (FACE) conditions and examined the effects of a large sink capacity on grain yield, its components, and growth-related traits under increased atmospheric CO_2_ concentrations. Under ambient conditions, CSSL-*GN1* and NIL-*APO1* exhibited a similar grain yield to Koshihikari, as a result of the trade-off between increased spikelet number and reduced grain filling. However, under FACE conditions, CSSL-*GN1* and NIL-*APO1* had an equal or a higher grain yield than Koshihikari because of the higher number of spikelets and lower reduction in grain filling. Thus, the improvement of source activity by increased atmospheric CO_2_ concentrations can lead to enhanced grain yield in rice lines that have a large sink capacity. Therefore, introducing alleles that increase sink capacity into conventional varieties represents a strategy that can be used to develop high-yielding varieties under increased atmospheric CO_2_ concentrations, such as those predicted in the near future.

## Introduction

Crop growth and yield are affected by global changes in the environment, such as increasing atmospheric carbon dioxide (CO_2_) concentrations and air temperature^1^. The atmospheric CO_2_ concentration has increased steadily from 280 μmol mol^−1^ before the Industrial Revolution, to 400 μmol mol^−1^ in 2015^2^, and is projected to continue to increase over the course of this century.

The world’s population is estimated to reach 9.1 billion people by 2050^3^. To feed such a large number of people, global food production must be increased substantially. Rice (*Oryza sativa* L.) is eaten by nearly half of the world’s population and is a staple food for most population^4^. Because arable land for rice is limited, improving rice yield per unit area is essential to resolve global food issues. However, the increased rice yield per unit area has been reduced in recent years throughout the world^5^.

Terrestrial plants containing a C_3_ photosynthetic pathway, including rice, are positively influenced by increased atmospheric CO_2_ through photosynthetic rates and water-use efficiency^6, 7^. To produce high grain yield in rice, a large sink capacity is needed, as well as the ability to produce high levels of carbohydrates and to translocate them to the sink. Rice grain yield is thought to be improved in response to rising atmospheric CO_2_ concentrations through an increased number of spikelets per square meter^8–12^. High-yielding varieties with a large sink capacity (spikelet number per square meter × single grain weight) have a higher grain-yield response to increased CO_2_ concentrations than conventional varieties with a general sink capacity^11^. Furthermore, there is a positive correlation between grain-yield response to increased CO_2_ concentrations and sink capacity. However, such high-yielding varieties with a large sink capacity generally possess other traits that allow them to produce a high grain yield. Therefore, direct evidence regarding the relationship between grain-yield response and increased CO_2_ concentration, and sink capacity is lacking.

Recent progress in rice genomics has facilitated genetic analyses of quantitative traits such as grain yield, and some genes regulating spikelet number per panicle of rice have been identified^13, 14^. One quantitative trait loci (QTL) *GN1a*, results in the production of more spikelets per panicle in the presence of an allele from an *indica* high-yielding variety than with that from a *japonica* conventional variety^13^. *GN1a* encodes the enzyme cytokinin oxidase/dehydrogenase (OsCKX2). In addition, *GN1b*, which is estimated to lie in the vicinity of *GN1a*, may also increase spikelet number per panicle with the allele from an indica high-yielding variety. Similarly, an allele of *APO1* derived from an *indica* high-yielding variety was found to produce a higher spikelet number per panicle than that from a *japonica* conventional variety^14^. However, in some field experiments performed under ambient conditions, near-isogenic lines (NIL) or chromosome segment substitution lines (CSSL) possessing such favorable alleles in the *japonica* genetic background did not have a higher grain yield due to the lower percentage of filled spikelets and 1000-grain weight^15, 16^, suggesting a lack of source activity relative to their large sink capacity.

To date, several laboratory and chamber experiments investigating the plant growth response to increased atmospheric CO_2_ concentrations have been conducted and reported^17^. However, plant growth responses in the laboratory or under chamber conditions may differ from those observed under field conditions. Free-air CO_2_ enrichment (FACE) experiments represent a promising method to grow plants at controlled levels of elevated CO_2_ under fully open-air field conditions^7^ in order to investigate yield response to CO_2_ increases.

Increased atmospheric CO_2_ concentrations lead to an increase in the dry matter yield of rice^10, 11^, indicating that source activity is raised. A previous rice FACE study showed that a variety Takanari with a large sink capacity is higher yielding and more responsive to increased CO_2_ concentrations than a conventional cultivar Koshihikari^11^. To raise yield potentials under increased CO_2_ concentrations efficiently and effectively, we need to understand the effects of QTLs that can enhance sink capacity on yield performance in increased CO_2_ under open field conditions. Recently, a CSSL and a NIL were developed carrying *GN1* and *APO1* alleles, respectively, from Takanari in the Koshihikari genetic background^16^. We therefore hypothesized that *GN1* and *APO1* alleles, which produce a higher spikelet number per panicle, have a high grain yield as a result of enhanced source activity under increased atmospheric CO_2_ concentrations. To test this hypothesis, we grew a conventional variety, Koshihikari, and CSSL-*GN1* and NIL-*APO1* with the Koshihikari genetic background under FACE conditions and examined the effects of a large sink capacity on grain yield, its components, and growth related traits under increased atmospheric CO_2_ concentrations (200 μmol mol^−1^ above the ambient CO_2_). On the basis of the results, we consider whether introducing such alleles to conventional varieties represents an effective method of increasing grain yield under increased atmospheric CO_2_ concentrations, such as those predicted in the near future.

## Results

There were no significant interactions between year and CO_2_ concentration or among year, CO_2_ concentration, and genotype for grain yield, so data were combined over two years.

### Grain yield

Grain yield was affected by CO_2_ concentration and genotype. There was an interaction between CO_2_ concentration and genotype for grain yield (Table 1). In Koshihikari, grain yield did not differ between CO_2_ concentrations (FACE/ambient = 1.08). However, in CSSL-*GN1* and NIL-*APO1*, grain yield under FACE conditions was increased compared with that under ambient conditions (FACE/ambient = 1.21 and 1.19, respectively). Under ambient conditions, CSSL-*GN1* and NIL-*APO1* had almost the same grain yield as Koshihikari. In contrast, under FACE conditions, CSSL-*GN1* and NIL-*APO1* had an equal or a higher grain yield than Koshihikari.

**Table 1:** Mean grain yield, its components, and harvest index as affected by different CO_2_ concentrations and genotypes averaged for two years (2012 and 2013).

CO_2_ Concentration	Genotype	Grain Yield (g m^−2^)	Spikelet number (× 10^3^ m^−2^)	Panicle number (m^−2^)	Spikelet number (panicle^−1^)	Percentage of filled spikelets (%)	1000-grain weight (g)	Harvest Index
CO_2_ concentration (C)								
FACE		813	47.0	333	143	85.4	20.5	0.41
Ambient		702	44.4	326	138	77.6	20.6	0.40
Genotype (G)								
	Koshihikari	735b^†^	40.5c	358a	113c	86.5a	21.0a	0.39b
	CSSL-*GN1*	724b	51.3a	322b	160a	72.9b	19.4b	0.39b
	NIL-*APO1*	814a	45.3b	308b	147b	85.0a	21.1a	0.43a
C × G								
FACE	Koshihikari	763b	40.6	358	114	89.2	21.1	0.40b
	CSSL-*GN1*	791abA^‡^	53.4	328	163	78.0	19.1	0.40bA
	NIL-*APO1*	886aA	47.0	313	151	89.0	21.2	0.43a
Ambient	Koshihikari	708ab	40.3	359	113	83.9	21.0	0.39b
	CSSL-*GN1*	656bB	49.3	316	157	67.7	19.7	0.38cB
	NIL-*APO1*	743aB	43.5	304	144	81.0	21.1	0.42a
ANOVA								
CO_2_ concentration (C)		NS^¶^	NS	NS	NS	**	NS	§
Genotype (G)		**	**	**	**	**	**	**
C × G		*	NS	NS	NS	NS	**	§

*Significant at P < 0.05. **Significant at P < 0.01. ^†^Means within a column followed by the same lowercase letter do not differ significantly (P < 0.05). ^‡^Means within a column followed by the same lowercase letter do not differ significantly (P < 0.05) among genotypes for a given CO_2_ concentration. Means within a column followed by the same uppercase letter do not differ significantly (P < 0.05) between CO_2_ concentrations for a given genotype. ^§^Significant at P < 0.10. ^¶^Not significant at P < 0.10.

### Grain-yield components and harvest index

Grain-yield components relating to spikelet number were affected by CO_2_ concentration and genotype (Table 1). In all tested genotypes, spikelet number per square meter, panicle number per square meter, and spikelet number per panicle did not differ between CO_2_ concentrations. Under both CO_2_ concentrations, CSSL-*GN1* and NIL-*APO1* had a lower panicle number per square meter than Koshihikari, but had a much higher spikelet number per panicle. Consequently, CSSL-*GN1* and NIL-*APO1* had a higher spikelet number per square meter than Koshihikari.

Grain yield components relating to grain filling were influenced by CO_2_ concentration and genotype (Table 1). In all tested genotypes, the percentage of filled spikelets under FACE conditions was increased compared with that under ambient conditions. Under both CO_2_ concentrations, CSSL-*GN1* had low percentage of filled spikelets and 1000-grain weight relative to Koshihikari, whereas NIL-*APO1* had almost the same percentage and weight as Koshihikari.

There was an interaction between CO_2_ concentration and genotype for harvest index. Under ambient condition, CSSL-*GN1* had the lowest harvest index in all tested genotypes. However, under FACE condition, CSSL-*GN1* had a similar harvest index to Koshihikari. On the other hand, NIL-*APO1* had the highest harvest index in all tested genotypes consistently across two CO_2_ concentrations.

### Growth-related traits at heading

The effects of CO_2_ concentration and genotype on growth-related traits at heading were examined (Table 2). In all tested genotypes, DM weight, stem DM weight, and NSC concentration under FACE conditions were increased compared with those under ambient conditions. As a result, the NSC amount under FACE conditions was larger than that under ambient conditions. In addition, LAI did not differ between CO_2_ concentrations and among genotypes.

**Table 2:** Mean dry matter (DM) weight, nonstructural carbohydrate (NSC) concentration and its amount in the leaf sheaths plus stems, and leaf area index (LAI) at heading as affected by different CO_2_ concentrations and genotypes averaged for two years (2012 and 2013).

CO_2_ concentration	Genotype	DM weight (g m^−2^)	Stem DM weight (g m^−2^)	NSC concentration (g kg^−1^)	NSC amount (g m^−2^)	LAI
CO_2_ concentration (C)						
FACE		1152	713	370	264	4.33
Ambient		1038	609	332	203	4.67
Genotype (G)						
	Koshihikari	1069	674	366	248	4.37
	CSSL-*GN1*	1093	643	335	217	4.60
	NIL-*APO1*	1124	666	351	235	4.53
C × G						
FACE	Koshihikari	1127	731	380	277	4.12
	CSSL-*GN1*	1151	694	359	250	4.48
	NIL-*APO1*	1179	714	371	265	4.40
Ambient	Koshihikari	1011	617	353	219	4.62
	CSSL-*GN1*	1036	591	312	185	4.73
	NIL-*APO1*	1069	619	332	205	4.66
ANOVA						
CO_2_ concentration (C)		*	**	*	**	NS
Genotype (G)		NS^†^	NS	NS	NS	NS
C × G		NS	NS	NS	NS	NS

*Significant at P < 0.05. **Significant at P < 0.01. ^†^Not significant at P < 0.10.

### Growth-related traits at maturity

The effects of CO_2_ concentration and genotype on growth-related traits at maturity were examined (Table 3). In all tested genotypes, DM weight, stem DM weight, and NSC concentration under FACE conditions were increased compared with those under ambient conditions. There was an interaction between CO_2_ concentration and genotype for the NSC amount. In all tested genotypes, NSC amount was increased compared with that under ambient condition. Under ambient condition, the NSC amount did not differ among genotypes. However, under FACE condition, CSSL-*GN1* and NIL-*APO1* had a lower NSC amount than Koshihikari. In addition, ΔW did not differ between CO_2_ concentrations and among genotypes.

**Table 3:** Mean dry matter (DM) weight, nonstructural carbohydrate (NSC) concentration, and its amount in the leaf sheaths plus stems at maturity, and DM increase from heading to maturity (ΔW) affected by different CO_2_ concentrations and genotypes averaged for two years (2012 and 2013).

CO_2_ concentration	Genotype	DM weight (g m^−2^)	Stem DM weight (g m^−2^)	NSC concentration (g kg^−1^)	NSC amount (g m^−2^)	ΔW (g m^−2^)
CO_2_ concentration (C)						
FACE		1991	786	305	241	833
Ambient		1777	664	264	176	739
Genotype (G)						
	Koshihikari	1874	762a^†^	294	227a	805
	CSSL-*GN1*	1864	691b	286	199b	771
	NIL-*APO1*	1913	722ab	273	200b	790
C × G						
FACE	Koshihikari	1938	830	326	271aA^‡^	811
	CSSL-*GN1*	1982	738	296	219bA	831
	NIL-*APO1*	2051	789	294	233bA	872
Ambient	Koshihikari	1811	693	262	183B	800
	CSSL-*GN1*	1746	644	276	179B	711
	NIL-*APO1*	1775	654	252	166B	707
ANOVA						
CO_2_ concentration (C)		§	*	**	**	NS
Genotype (G)		NS^¶^	**	NS	*	NS
C × G		NS	NS	NS	§	NS

*Significant at P < 0.05. **Significant at P < 0.01. ^†^Means within a column followed by the same lowercase letter do not differ significantly (P < 0.05). ^‡^Means within a column followed by the same lowercase letter do not differ significantly (P < 0.05) among genotypes for a given CO_2_ concentration. Means within a column followed by the same uppercase letter do not differ significantly (P < 0.05) between CO_2_ concentrations for a given genotype. ^§^Significant at P < 0.10. ^¶^Not significant at P < 0.10.

### Relationships between agronomic traits in CSSL-*GN1* and NIL-*APO1*

Grain yield of  CSSL-*GN1* and NIL-*APO1* was positively correlated with the percentage of filled spikelets, NSC amount at heading, and ΔW grain yield, but not with spikelet number per square meter and 1000-grain weight (Table 4). Also, the percentage of filled spikelets was positively correlated with correlated with 1000-grain weight and NSC amount at heading, but not with spikelet number per square meter and ΔW.

**Table 4:** Pearson correlation (*r*) analysis for agronomic traits in CSSL*-GN1* and NIL*-APO1* in 2012 and 2013.

	Spikelet number (×10^3^ m^−2﻿﻿^)	Percentage of filled spikelets (%)	1000-grain weight (g)	NSC amount at heading (g m^−2^)	ΔW^†^ (g m^−2^)
Grain yield	−0.046	0.925***	0.429	0.874***	0.610^‡^
Percentage of filled spikelets	−0.375	—	0.611^‡^	0.762**	0.574

***Significant at P < 0.001. **Significant at P < 0.01. ^†^Dry matter increase from heading to maturity. ^‡^Significant at P < 0.10.

### Percentage of spikelet and spikelet weight at each position in the panicle

**Figure 1:**
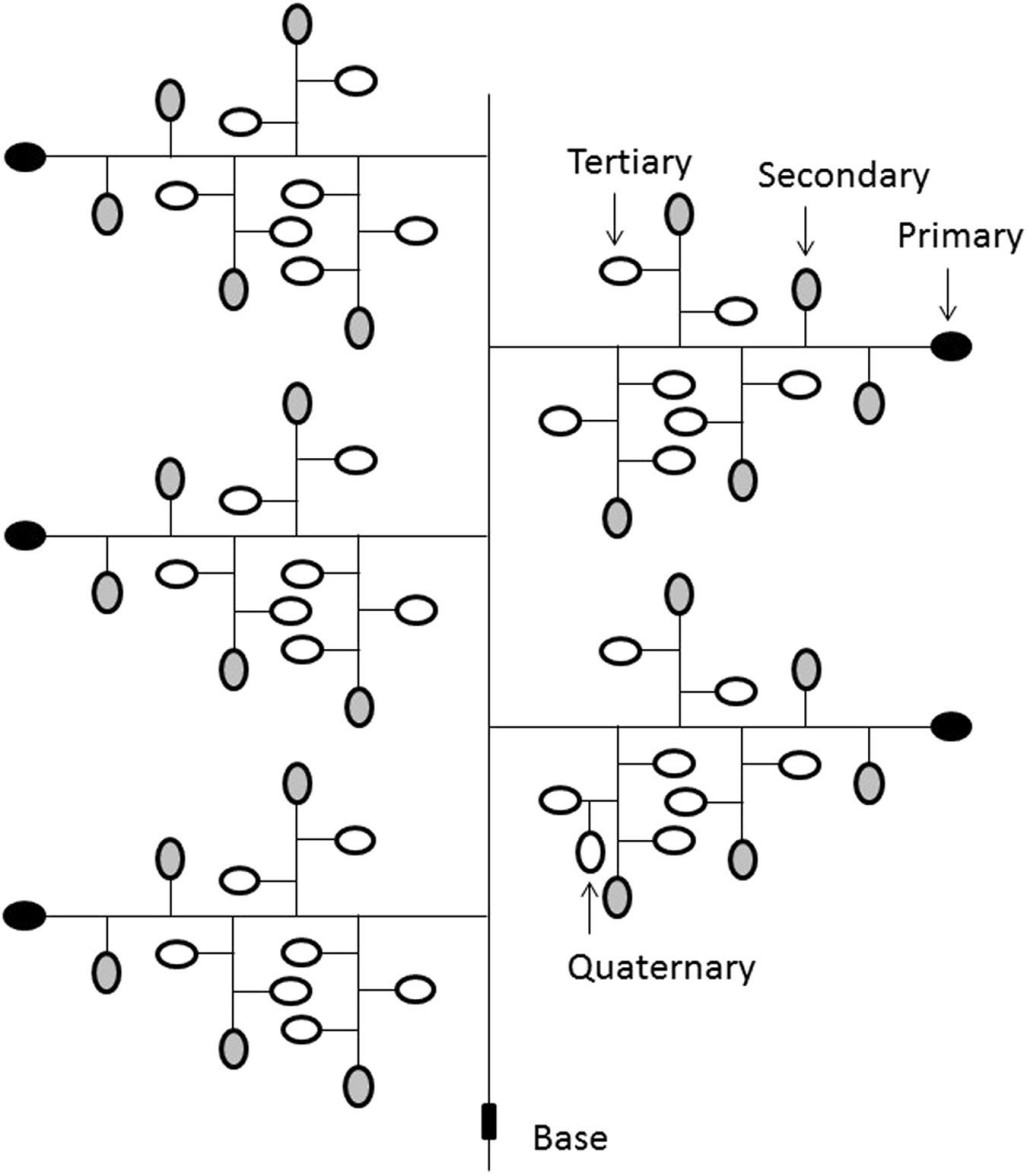
Panicle structure of rice.

The percentage of spikelets at each position in the panicle was affected by genotype but not by CO_2_ concentration (Fig. 1 and﻿ Table 5). Under both CO_2_ concentrations, CSSL-*GN1* and NIL-*APO1* had a lower percentage of primary and secondary spikelets than Koshihikari, but a higher percentage of tertiary spikelets.

**Table 5:** Mean percentage of spikelet and spikelet weight per spikelet at each position (primary, secondary, tertiary, and quaternary) in the panicles as affected by different CO_2_ concentrations and genotype averaged for two years (2012 and 2013).

CO_2_ concentration	Genotype	Percentage of spikelet				Spikelet weight			
		Primary (%)	Secondary (%)	Tertiary (%)	Quaternary (%)	Primary (mg spikelet^−1^)	Secondary (mg spikelet^−1^)	Tertiary (mg spikelet^−1^)	Quaternary (mg spikelet^−1^)
CO_2_ concentration (C)									
FACE		6.7	52.8	40.4	0.0	24.6	23.6	18.7	0.3
Ambient		6.9	53.6	39.6	0.0	23.9	23.3	15.5	0.0
Genotype (G)									
	Koshihikari	7.7a^†^	57.5a	34.8b	0.0	24.7a	24.5a	19.2a	0.0
	CSSL-*GN1*	6.2b	50.5b	43.3a	0.0	23.6b	21.9b	14.3b	0.0
	NIL-*APO1*	6.6b	51.5b	41.9a	0.1	24.5a	24.0a	17.8a	0.5
C × G									
FACE	Koshihikari	7.7	57.6	34.7	0.0	25.6aA^‡^	25.1	21.5	0.0
	CSSL-*GN1*	6.0	49.8	44.2	0.0	23.3b	21.8	15.1	0.0
	NIL-*APO1*	6.5	51.0	42.5	0.1	24.8a	24.0	19.4	1.0
Ambient	Koshihikari	7.7	57.4	34.8	0.0	23.8B	23.9	16.9	0.0
	CSSL-*GN1*	6.3	51.3	42.5	0.0	23.9	22.1	13.4	0.0
	NIL-*APO1*	6.7	52.1	41.3	0.0	24.1	24.1	16.1	0.0
ANOVA									
CO_2_ concentration (C)		NS^¶^	NS	NS	NS	NS	NS	*	NS
Genotype (G)		**	**	**	NS	§﻿^§^	*	*	NS
C × G		NS	NS	NS	NS	§	NS	NS	NS

*Significant at P < 0.05. **Significant at P < 0.01. ^†^Means within a column followed by the same lowercase letter do not differ significantly (P < 0.05). ^‡^Means within a column followed by the same lowercase letter do not differ significantly (P < 0.05) among genotypes for a given CO_2_ concentration. Means within a column followed by the same uppercase letter do not differ significantly (P < 0.05) between CO_2_ concentrations for a given genotype. ^§^Significant at P < 0.10. ^¶^Not significant at P < 0.10.

Spikelet weight at each position in the panicle was influenced by CO_2_ concentration and genotype (Fig. 1 a﻿nd ﻿Table 5). There was an interaction between CO_2_ concentration and genotype for primary spikelet weight. In Koshihikari, the primary spikelet weight under FACE condition was increased compared with that under ambient condition. However, in CSSL-*GN1* and NIL-*APO1*, the primary spikelet weight did not differ between CO_2_ concentrations. Under ambient conditions, primary spikelet weight did not differ among genotypes. However, under FACE conditions, CSSL-*GN1* had a low primary spikelet weight relative to Koshihikari, whereas NIL-*APO1* had a similar primary spikelet weight to Koshihikari. In all tested genotypes, the tertiary spikelet weight under FACE conditions was increased compared with that in ambient. Under both CO_2_ concentrations, CSSL-*GN1* had low secondary and tertiary spikelet weights relative to Koshihikari, whereas NIL-*APO1* had almost the same secondary and tertiary spikelet weights as Koshihikari.

### Spikelet number and weight per square meter at each position in the panicle

Spikelet number at each position in the panicle per square meter were affected by CO_2_ concentration and genotype (Fi﻿g. 1﻿ and﻿ Table 6). In all tested genotypes, the tertiary spikelet number per square meter under FACE conditions was increased compared with that under ambient conditions. Under both CO_2_ concentrations, CSSL-*GN1* had a higher secondary spikelet number than Koshihikari, whereas NIL-*APO1* had almost the same number as Koshihikari. Similarly, CSSL-*GN1* had the highest tertiary spikelet number, followed by NIL-*APO1* and Koshihikari.

**Table 6:** Mean spikelet number and spikelet weight per square meter at each position (primary, secondary, tertiary, and quaternary) in the panicles as affected by different CO_2_ concentrations and genotypes averaged for two years (2012 and 2013).

CO_2_ concentration	Genotype	Spikelet number				Spikelet weight			
		Primary (×10^3^ m^−2^)	Secondary (×10^3^ m^−2^)	Tertiary (×10^3^ m^−2^)	Quaternary (×10^3^ m^−2^)	Primary (g m^−2^)	Secondary (g m^−2^)	Tertiary (g m^−2^)	Quaternary (g m^−2^)
CO_2_ concentration (C)									
FACE		3.1	24.6	19.2	0.0	77	579	350	0.0
Ambient		3.0	23.7	17.7	0.0	72	552	271	0.0
Genotype (G)									
	Koshihikari	3.1	23.3b^†^	14.1c	0.0	77	569	271	0.0
	CSSL-*GN1*	3.2	26.0a	22.2a	0.0	74	570	319	0.0
	NIL-*APO1*	3.0	23.2b	19.0b	0.0	72	558	341	0.0
C × G									
FACE	Koshihikari	3.1	23.4	14.2	0.0	80	584	305	0.0
	CSSL-*GN1*	3.2	26.7	23.6	0.0	75	581	355	0.0
	NIL-*APO1*	3.0	23.9	20.0	0.1	75	572	389	0.0
Ambient	Koshihikari	3.1	23.2	14.1	0.0	74	554	237	0.0
	CSSL-*GN1*	3.1	25.3	20.9	0.0	74	559	283	0.0
	NIL-*APO1*	2.9	22.6	18.0	0.0	69	544	293	0.0
ANOVA									
CO_2_ concentration (C)		NS§	NS	‡	NS	NS	NS	‡	NS
Genotype (G)		‡	*	**	NS	‡	NS	NS	NS
C × G		NS	NS	NS	NS	NS	NS	NS	NS

*Significant at P < 0.05. **Significant at P < 0.01. ^†^Means within a column followed by the same lowercase letter do not differ significantly (P < 0.05). ^‡^Significant at P < 0.10. ^§^Not significant at P < 0.10.

Spikelet weight at each position in the panicle per square meter was influenced by CO_2_ concentration, but not by genotype (Fig. 1 and﻿ Table 6). In all genotypes, tertiary spikelet weight under FACE conditions was increased compared with that under ambient conditions.

## Discussion

The results of previous studies have indicated that CSSL and NIL, carrying *GN1* or *APO1* alleles derived from high-yielding *indica* varieties, did not exhibit high grain yield despite their large sink capacity^15, 16^. In the present study, CSSL-*GN1* and NIL-*APO1* had almost the same grain yield as Koshihikari under ambient conditions because of the trade-off between increased spikelet number and reduced grain filling (Table 1). Furthermore, CSSL-*GN1* had a lower grain yield than NIL-*APO1*. The low grain yield of CSSL-*GN1* may result from its extremely large sink capacity. These results confirmed that high source activity is required to increase grain yield in rice lines that have a large sink capacity.

In contrast to this, under FACE conditions, CSSL-*GN1* and NIL-*APO1* had an equal or a higher grain yield than Koshihikari because of the higher number of spikelets and lower reduction in grain filling. In addition, although the effect of increased atmospheric CO_2_ concentration on spikelet number was not clear in the present study (Table 1), spikelet number per square meter was reportedly increased by increased atmospheric CO_2_ concentrations^8–12, 18^.

Grain carbohydrates are derived from both accumulated carbohydrates (i.e., NSC) in the leaf sheaths plus stems at heading and photosynthetic products during grain filling (i.e., ΔW)^19^. The contributions of NSC at heading and ΔW to grain carbohydrates range from 0 to 40% and 60 to 100%, respectively, under most weather conditions during grain filling. However, NSC at heading is considered to be important to stabilize grain yield under unfavorable weather conditions during grain filling, because it compensates for the reduction in ΔW^20–22^. The NSC amount at heading in the present study were increased by increased atmospheric CO_2_ concentrations in all tested genotypes (Tables 2). The large amount of NSC at heading resulted from the stem DM weight, and NSC concentration at heading. The results of a previous study indicated that grain yield was closely related to crop growth rate (CGR) during the late reproductive period^23^. The high CGR led to the production of a high number of spikelets per square meter and a high level of NSC amount at heading, which was associated with the rapid translocation of NSC to the panicle during the initial period of grain filling. In the present study, the grain yield of CSSL-*GN1* and NIL-*APO1* was related to the percentage of filled spikelets and NSC amount at heading, and the percentage of filled spikelets was related to the NSC amount at heading (Table 4). In addition, the results of a recent study revealed that spikelet sterility of varieties with a high number of spikelets was caused by a lack of assimilate supply around flowering^24^. Thus, improved source activity in response to increased atmospheric CO_2_ concentration can enhance grain yield in rice lines that have a large sink capacity.

In the present study, in all tested genotypes, NSC amount at maturity was increased by increased atmospheric CO_2_ concentrations (Table 3), whereas harvest index was not decreased by atmospheric CO_2_ concentrations in CSSL-*GN1* (Table 1). Under ambient condition, CSSL-*GN1* had the lowest harvest index in all tested genotypes. However, under FACE condition, CSSL-*GN1* had a similar harvest index to Koshihikari. Varieties with a small sink capacity have a large amount of NSC at the late grain filling stage^25, 26^. Hence, under FACE conditions, Koshihikari may not be able to translocate a large amount of carbohydrates to the panicle due to its limited sink capacity, whereas CSSL-*GN1* and NIL-*APO1* are able to this due to their large sink capacity.

Primary spikelets have an advantage for grain filling over secondary spikelets^27^, which is advantageous for grain filling compared with tertiary and quaternary spikelets. In all tested genotypes, the number of tertiary spikelets per square meter in the present study was increased by increased atmospheric CO_2_ concentrations (Fig. 1 and Table 6). Under both CO_2_ concentrations, CSSL-*GN1* and NIL-*APO1* had a higher percentage of tertiary spikelets and more tertiary spikelets per square meter than Koshihikari (Tables 5 and 6), indicating that CSSL-*GN1* and NIL-*APO1* possess more disadvantaged spikelets for grain filling under FACE conditions. However, in CSSL-*GN1* and NIL-*APO1* as well as in Koshihikari, the tertiary spikelet weight per spikelet and the spikelet weight per square meter was increased by increased atmospheric CO_2_ concentration. Therefore, in CSSL-*GN1* and NIL-*APO1*, increased atmospheric CO_2_ concentrations may increase the tertiary grain weight per spikelet in spite of their large tertiary spikelet number per square meter resulting in an increased percentage of filled spikelets. In a recent study, Koshihikari and Takanari were compared, and Takanari, which has a higher number of spikelets, showed a substantial increase in grain weight of inferior spikelets under FACE conditions, while the weight of superior spikelets was not affected under those conditions^28^. The results of the present study confirm that the increased weight of the inferior spikelets could be enhanced by increased source capacity, and could contribute to a greater yield response to elevated CO_2_ conditions. It is worth noting, however, that the source capacity for Takanari was also significantly greater than that for Koshihikari under both current and future CO_2_ conditions^29^, suggesting that an increase in source capacity is also needed to meet the increased demand of grain for carbon.

To resolve global food issues, there is a need to develop high-yielding varieties that can adjust to the future environment. Recently developed high-yielding varieties in Japan are divided into two groups, inbred varieties from *indica* and *indica-japonica* varieties (i.e., pureline varieties originated from the cross of *indica* and *japonica* parents). *Indica* high-yielding varieties developed in Japan have higher percentage of filled spikelets than *indica-japonica* high-yielding varieties^30, 31^, because they have higher grain weight of tertiary spikelet^30^. This suggests that they are able to produce high levels of carbohydrates and translocate them to their sink. *Indica-japonica* high-yielding varieties have a higher sink capacity than *indica* high-yielding varieties developed in Japan^31^. Thus, such varieties are expected to produce a high grain yield under the increased atmospheric CO_2_ concentrations that may occur in the near future.

Lodging reduces grain yield as a result of self-shading and reduced canopy photosynthesis^32^. The results of a previous report indicated that lodging was increased by increasing panicle weight under ambient conditions, but was alleviated under FACE conditions^33^. Consequently, to develop varieties which can contribute a stable and high production of rice under increased atmospheric CO_2_ concentrations in the near future, introducing alleles to conventional varieties that enhance sink capacity represents a useful strategy, because the risk of lodging as a results of increasing panicle weight is relatively low.

## Materials and Methods

### Experimental design and crop management

The study was conducted in 2012 and 2013 on a Fluvisol, which is typical of alluvial areas, at the Tsukubamirai free-air CO_2_ enrichment field (35°58′N, 139°60′E, 10 m above sea level), Tsukubamirai, Ibaraki, Japan. Rice was grown previously in the field was rice in both years. Treatments included two atmospheric CO_2_ concentrations and three genotypes (one variety and two lines), which were arranged as a split-plot experiment with four replicates in a randomized complete block design. The main plot and subplot were atmospheric CO_2_ concentration and genotype, respectively. The mean temperature and solar radiation recorded during the cropping season in 2012 and 2013 were higher than those in a normal year (Table 7).

**Table 7:** Mean temperature and solar radiation at the Tsukubamirai free-air CO_2_ enrichment (FACE) facility, Tsukubamirai, Ibaraki, Japan, during the 2012 and 2013 crop seasons.

Month	Stage of month	Mean temperature			Solar radiation		
		2012 (°C)	2013 (°C)	Normal^†^ (°C)	2012 (MJ m^−2^ d^−1^)	2013 (MJ m^−2^ d^−1^)	Normal (MJ m^−2^ d^−1^)
May	early						
	middle						
	late	19.2	20.1	17.9	21.4	20.1	18.4
June	early	19.9	20.1	19.4	18.5	22.7	17.8
	middle	19.8	22.5	20.2	15.2	10.5	15.5
	late	19.8	21.3	20.9	20.6	17.3	13.4
July	early	22.9	25.6	22.5	17.6	20.0	15.2
	middle	25.8	25.1	23.9	19.6	21.1	15.3
	late	25.8	24.5	25.2	19.4	15.8	17.8
August	early	26.2	26.6	25.8	20.5	19.2	18.1
	middle	26.9	28.4	25.5	17.9	22.6	17.3
	late	27.4	25.9	25.1	21.5	17.1	15.8
September	early	25.4	25.0	24.0	16.1	13.9	14.4
	middle	25.9	23.3	22.0	15.5	16.7	12.3
	late						

^†^30-yr average (1981–2010) recorded at the nearest weather station Tateno.

The following variety and lines were used: Koshihikari, which is a conventional variety in Japan; a CSSL carrying the *GN1* region from a high-yielding variety, Takanari, in the Koshihikari genetic background (CSSL-*GN1*); and a NIL carrying a favorable allele of *APO1* from Takanari in the Koshihikari background (NIL-*APO1*). CSSL-*GN1* was developed by repeated backcrossing with Koshihikari and marker-assisted selection^16^. NIL-*APO1* was developed in the same way. The selection of the alleles of Takanari were made based on our previous FACE experiment that Takanari showed a greater yield response with greater sink capacity^11^.

Germinated seeds were sown in nursery boxes in late April. Seedlings were transplanted by hand into the paddy field in late May at a density of 22.2 hills m^−2^ (three seedlings per hill, 30 cm wide × 15 cm long). About a month before transplanting, plots received 100 kg ha^−1^ P_2_O_5_, and 100 kg ha^−1^ K_2_O in the form of synthetic fertilizer broadcast by hand. One week before transplanting, plots received 120 kg ha^−1^ N in the form of synthetic fertilizer (urea/LP100/LP140, 1:2:1) broadcast by hand, with the fertilizer incorporated into the soil by puddling and leveling with a harrow. One month before heading, plants received 40 kg ha^−1^ N in the form of synthetic fertilizer (LP40) broadcast by hand. LP40, LP100, and LP140 (JCAM AGRI. Co., Ltd., Tokyo, Japan) are controlled-release fertilizers, which release 80% of the total N content at a uniform rate up to 40, 100, and 140 days after application, respectively, at 20–30 °C. After trimming, each plot was 1.5-m wide × 2.25-m long. To prevent lodging, a polyethylene netting with a horizontal of 30 cm × 30 cm about 70 cm above the soil surface was installed in each plot. Weeds and pests were controlled with biocides such as those described by Hasegawa *et al*.^11^.

### CO_2_ control

The atmospheric CO_2_ concentration was controlled as described by Nakamura *et al*.^34^. The average atmospheric CO_2_ concentration ± day-to-day standard deviations during the crop season in FACE plots was 578 ± 15.7 μmol mol^−1^ in 2012 and 576 ± 15.5 μmol mol^−1^ in 2013, and in ambient plots was 383 ± 11.2 μmol mol^−1^ in 2012 and 383 ± 11.4 μmol mol^−1^ in 2013.

### Sampling and measurements

At heading (Zadoks code 59)^35^ (early August), plants from 0.405 m^2^ (nine hills) were sampled. Two hills with an average panicle number were selected and separated into green leaf blades, dead leaf blades, leaf sheaths plus stems, and panicles. After the area of green leaf blades was measured with a leaf area meter (AAM-9, Hayashi Denko, Tokyo, Japan), each plant part was dried at 80 °C in a ventilated oven for 2 days with the plants of the remaining hills to determine their dry weight. The dried samples were ground to a powder with a vibrating sample mill (TI-1001, CMT, Co., Ltd., Tokyo, Japan) in order to measure nonstructural carbohydrate (NSC) concentrations. Concentrations of NSCs in the leaf sheaths plus stems were determined as described by Ohnishi and Horie^36^.

At maturity (Zadoks code 92)^35^ (mid-September), plants from 0.810 and 0.855 m^2^ (18 and 19 hills) in 2012 and 2013, respectively, were sampled. Two hills were selected with an average panicle number and were separated into leaf blades, leaf sheaths plus stems, and panicles. Their NSC concentrations were determined as described. The 15 hills were air-dried until they reached a constant weight. The panicles were counted, and the air-dried plants were threshed. Half of the rough rice grains were dehusked to determine the grain weight and the 1000-grain weight. Grain numbers with a thickness of 1.6 mm or more making up 20 g were counted with a multi auto counter (KC-10, Fujiwara Seisakusho, Tokyo, Japan), and the 1000-grain weights were calculated from this value. Grain yield and 1000-grain weight were corrected based on a 150 g kg^−1^ moisture concentration. Approximately 100 g of rough rice grains were counted with a multi auto counter (KC-10, Fujiwara Seisakusho, Tokyo, Japan) to determine the spikelet numbers. The remaining two hills were harvested and air-dried until they reached a constant weight. The spikelet numbers at each position (primary, secondary, tertiary, and quaternary) in the three panicles with greater weights were recorded as described in Fig. 1, and then the weights of the spikelets were determined.

### Statistical analysis

Statistical analyses were performed using a general linear model in SPSS (SPSS 17.0, SPSS Inc., Chicago, IL, USA). CO_2_ concentration and genotype were considered as fixed effects. Year and replication were considered as random effects. Analysis of variance (ANOVA) was conducted to test the effects of CO_2_ concentration and genotype on yield, its components, and panicle structure. Significant treatment effects (P < 0.05) were explored using Fisher’s protected least significant difference (LSD).
